# Mechanisms of oligodendrocyte regeneration from ventricular-subventricular zone-derived progenitor cells in white matter diseases

**DOI:** 10.3389/fncel.2013.00275

**Published:** 2013-12-26

**Authors:** Takakuni Maki, Anna C. Liang, Nobukazu Miyamoto, Eng H. Lo, Ken Arai

**Affiliations:** Neuroprotection Research Laboratory, Departments of Radiology and Neurology, Massachusetts General Hospital and Harvard Medical SchoolCharlestown, MA, USA

**Keywords:** oligodendrogenesis, oligodendrocyte precursor cells, vascular dementia, multiple sclerosis, demyelination, subventricular zone, neural stem/progenitor cells

## Abstract

White matter dysfunction is an important part of many CNS disorders including multiple sclerosis (MS) and vascular dementia. Within injured areas, myelin loss and oligodendrocyte death may trigger endogenous attempts at regeneration. However, during disease progression, remyelination failure may eventually occur due to impaired survival/proliferation, migration/recruitment, and differentiation of oligodendrocyte precursor cells (OPCs). The ventricular-subventricular zone (V-SVZ) and the subgranular zone (SGZ) are the main sources of neural stem/progenitor cells (NSPCs), which can give rise to neurons as well as OPCs. Under normal conditions in the adult brain, the V-SVZ progenitors generate a large number of neurons with a small number of oligodendrocyte lineage cells. However, after demyelination, the fate of V-SVZ-derived progenitor cells shifts from neurons to OPCs, and these newly generated OPCs migrate to the demyelinating lesions to ease white matter damage. In this mini-review, we will summarize the recent studies on extrinsic (e.g., vasculature, extracellular matrix (ECM), cerebrospinal fluid (CSF)) and intrinsic (e.g., transcription factors, epigenetic modifiers) factors, which mediate oligodendrocyte generation from the V-SVZ progenitor cells. A deeper understanding of the mechanisms that regulate the fate of V-SVZ progenitor cells may lead to new therapeutic approaches for ameliorating white matter dysfunction and damage in CNS disorders.

## Introduction

During embryogenesis and development, germinal zones form stem cell niches, where multi-potential progenitor cells generate new neurons, astrocytes, and oligodendrocyte lineage cells. In the adult brain, the ventricular-subventricular zone (V-SVZ) of the lateral ventricle and the subgranular zone (SGZ) in the dentate gyrus of hippocampus retain neural stem/progenitor cells (NSPCs) to form the largest germinative areas for new neurons and glial cells (Gonzalez-Perez and Alvarez-Buylla, 2011; Ihrie and Alvarez-Buylla, 2011; Falcao et al., 2012). In addition, recent studies suggest that NSPCs also reside in the nonconventional zones outside of the V-SVZ and SGZ, such as the cerebral cortex (Nakagomi et al., 2009; Ohira et al., 2010), white matter (Nunes et al., 2003), and pia mater (Nakagomi et al., 2011, 2012). Among these germinative areas, the V-SVZ generates the most abundant number of stem cells in the adult brain that are capable of migrating to a long distance.

NSPCs play important roles in many CNS diseases as endogenous recovery mechanisms in injured brains areas (Ohab et al., 2006; Curtis et al., 2007, 2012; Nait-Oumesmar et al., 2007, 2008; Bedard et al., 2010; Lazarov and Marr, 2010; Ekonomou et al., 2011). Although NSPC responses are often thought to represent attempts to ameliorate neuronal loss in gray matter, emerging data now suggest that NSPCs may also be involved in endogenous recovery mechanisms in white matter. White matter dysfunction occurs in a wide spectrum of neurodegenerative conditions including multiple sclerosis (MS) and vascular dementia. Within damaged white matter areas, the fate of NSPCs may shift from neurons to oligodendrocyte lineage cells in order to compensate for oligodendrocyte death and myelin loss. The precise mechanisms underlying the fate determination are still mostly unknown. However, several factors have been proposed as key modulators in promoting the NSPC differentiation into oligodendrocyte lineage cells. In this mini-review, we will summarize extrinsic and intrinsic factors that regulate the fate and behavior of NSPCs in the V-SVZ under normal and diseased conditions.

## Oligodendrocyte generation from neural stem/progenitor cells (NSPCs)

Oligodendrocytes, one of the major glial cells in the CNS, produce a lipid-rich membrane called myelin. Each oligodendrocyte can enwrap up to 60 axonal segments, thereby enabling fast and salutatory nerve impulse conduction (Baumann and Pham-Dinh, 2001). During development, oligodendrocyte precursor cells (OPCs) are first generated in the germinal zones where they will proliferate. They then migrate to both grey and white matter areas where most will differentiate into mature oligodendrocytes and form myelin sheaths. Although myelinated tracts are formed early in life, renewal of myelin/oligodendrocyte continues throughout adult life (Paus et al., 1999; Dimou et al., 2008; Young et al., 2013). In addition, myelin in the adult CNS maintain some plasticity in response to changes in neural activity (Scholz et al., 2009) and brain injury (Nait-Oumesmar et al., 2008).

Under normal conditions in the adult brain, most V-SVZ progenitor cells give rise to neuronal linage cells. They migrate along the rostral migratory stream (RMS) to the olfactory bulbs, where they terminate and differentiate into mature interneurons (Gonzalez-Perez and Alvarez-Buylla, 2011; Ihrie and Alvarez-Buylla, 2011; Falcao et al., 2012). Oligodendrocytes can also be generated from V-SVZ cells in the adult brain, and newly generated OPCs migrate towards the corpus callosum and the white matter tracts of striatum and fimbria fornix (Menn et al., 2006). However, the ratio of V-SVZ progenitor cells differentiating into oligodendrocyte linage cells decrease after early postnatal period (Gonzalez-Perez and Alvarez-Buylla, 2011). Interestingly, in the V-SVZ area, neuronal and oligodendroglial progenies constitute separate lineages under physiological conditions. Using continuous live imaging and single-cell tracking of NSPCs, Ortega et al. (2013) have demonstrated that a single NSPC and its offsprings in the subventricular zone (SVZ) cannot show both neuronal and oligodendroglial progenies (Ortega et al., 2013). Furthermore, the adult SVZ is highly regionalized. The neuronal progeny of distinct identity is generated at different areas along the dorsoventral and rostrocaudal axes (Hack et al., 2005; Merkle et al., 2007). In addition, clones fated to generate oligodendrocytes are prevalent in NSPCs isolated from dorsolateral SVZ. On the contrary, ventrolateral SVZ regions consist of both neuronal and astroglial progenies with few oligodendroglial progeny (Costa et al., 2011; Ortega et al., 2011, 2013).

V-SVZ progenitor cells in the adult brain show some lineage plasticity under pathological conditions. After CNS damage, a number of progenitors migrate out of the RMS to the injured site. The fate of these progenitor cells can be dynamically altered according to the disease type. The fate of V-SVZ progenitor cells can shift from NSPCs to OPCs after demyelination, and these newly generated OPCs proliferate and migrate to the lesion areas (Nait-Oumesmar et al., 1999; Picard-Riera et al., 2002; Jablonska et al., 2010; Gonzalez-Perez and Alvarez-Buylla, 2011). In a model of experimental autoimmune encephalomyelitis (EAE), enhanced proliferation and migration of SVZ NSPCs are observed, and these mobilized cells give rise to oligodendrocytes and astrocytes without neurons in the injured white matter (Picard-Riera et al., 2002). In addition, demyelination would change the fate of glutamic acid decarboxylase 65 (GAD65)/doublecortin (Dcx)-expressing NSPCs derived from the adult SVZ to generate oligodendrocytes, rather than neurons, in corpus callosum (Jablonska et al., 2010). This process may restore developmental myelination to some extent; NSPCs that generate oligodendrocytes migrate from SVZ to developing white matter, where they stop dividing to differentiate and myelinate axons (John et al., 2002; Jablonska et al., 2010).

**Figure 1:**
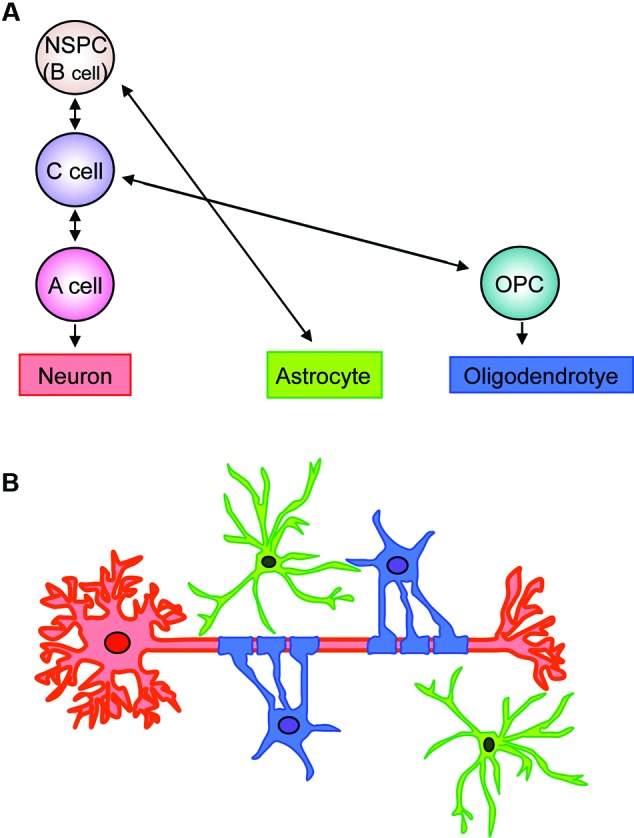
**Schematic of fate of neuronal, astroglial, oligodendrocytic lineage cells in V-SVZ**. **(A)** Type B cells retain neuroepithelial trait and function as NSPCs in the V-SVZ. Type B cells slowly divide and give rise to rapidly dividing type C cells (IPCs), which generate neuroblasts (type A cells). The type B cells can also generate astrocytes and Olig2-expressing type C cells. The Olig2-expressing type C cells give rise to highly migratory OPCs, which differentiate into myelinating oligodendrocytes. **(B)** Neurons, astrocytes, and oligodendrocytes, derived from V-SVZ NSPCs (type B cells), interact with each other to maintain proper neural function. Red: neuron, green: astrocyte, blue: oligodendrocyte.

Past studies have extensively examined the process of NSPC differentiation into oligodendrocyte lineage cells in the V-SVZ area (Figure 1). The V-SVZ contains a subpopulation of cells with astroglial properties (type B cells) that retain neuroepithelial trait and function as NSPCs. Type B cells slowly divide and give rise to rapidly dividing intermediate progenitor cells (IPCs) or transient amplifying progenitors (type C cells), which divide further to generate neuroblasts (type A cells). Although at a lower population, type B cells can also generate Olig2-expressing type C cells that give rise to highly migratory OPCs. These OPCs leave the V-SVZ and migrate to the corpus callosum and the white matter tract in the striatum and fimbria fornix (Menn et al., 2006; Gonzalez-Perez and Alvarez-Buylla, 2011; Ihrie and Alvarez-Buylla, 2011; Falcao et al., 2012; Fuentealba et al., 2012).

**Figure 2:**
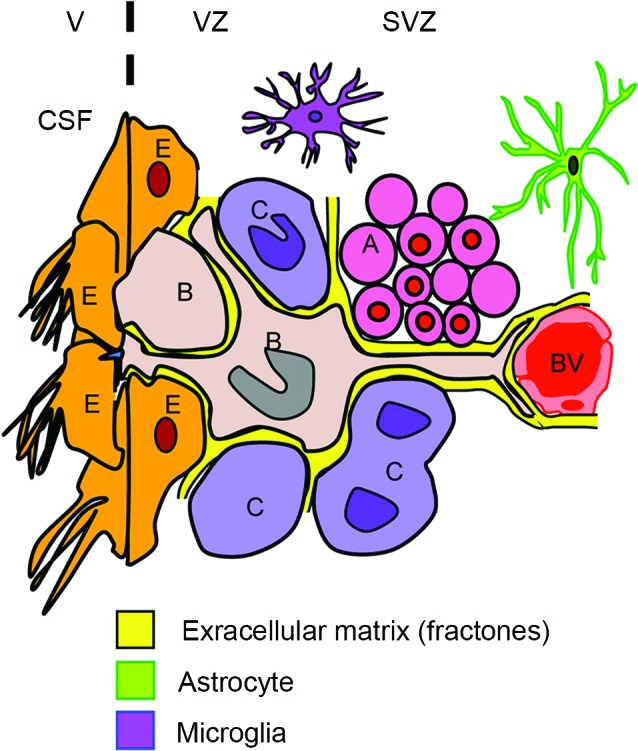
**Schematic of interplay for SVZ cells**. The subventricular zone (SVZ) and ventricular zone (VZ) are lining the lateral ventricles (V) in the brain. Type B cells (B) contact the ventricle (V) containing CSF through specialized apical processes. The processes contain a single primary cilium, which is surrounded by a rosette of ependymal cells (E) with large apical surfaces forming pinwheel-like structures. On the other side, the type B cells have long basal processes with specialized endings that frequently contact BV. The type B cells also contact their progeny, i.e., type C cells (C) and the chains of migrating type A neuroblasts (A). The V-SVZ includes ECM (fractones) that contacts all the cell types including BV, microglia, and astrocytes in this region.

NSPCs in the V-SVZ display diverse interactions with their neighboring environments (Falcao et al., 2012; Fuentealba et al., 2012; Figure 2). On one side of V-SVZ, type B cells are surrounded by multiciliated non-dividing ependymal cells, which form pinwheel-like structures on the ventricular surface. These cells are in direct contact with the cerebrospinal fluid (CSF) by a short non-motile primary cilium that extends towards the ventricle. On the other side, type B cells interact with the extensive network of blood vessels (BV) with a long basal process. Type B cells also attach to type C cells and chains of young neurons (type A cells) by the extracellular matrix (ECM). Proliferating type C cells are closely located to their progenitors, and are also often in close proximity to BV (Shen et al., 2008). Type B cells interact with one another by gap and adherens junctions, the same as ependymal cells (Mirzadeh et al., 2008). Furthermore, the adult V-SVZ possesses a highly organized basement membrane, which is absent in other areas of the brain (see the Section Extracellular Matrix).

Overall, the V-SVZ is poised to receive informational inputs via cell-cell and cell-matrix contacts. The integration of these multifaceted external cues (e.g., extracellular signals from the vasculature, ECM, and the cerebrospinal fluid) to intrinsic factors leads to the determination of the fate and behavior of each cell lineage. In the next section, we will discuss the components that can potentially shift the fates and behaviors of NSPCs towards oligodendrocyte lineage cells.

## Extrinsic factors that promote oligodendrocyte generation

### Vasculature

The vasculature is an integral component of the V-SVZ stem cell niche that possesses specialized properties in regulating stem cell proliferation and regeneration (Shen et al., 2008; Tavazoie et al., 2008). Endothelial cells can secrete factors that contribute to stem cell self-renewal or proliferation. Co-culture of endothelial cells with NPSCs enhance the *in-vitro* neurosphere generation from embryonic progenitors (Shen et al., 2004). NSPCs were shown to have direct coupling with cerebral endothelial cells (Teng et al., 2008), and various kinds of perivascular regulators, including growth factors, purinergic signaling, nitric oxide signaling, and chemokines, contribute to cell genesis and fate determination in the V-SVZ (Goldman and Chen, 2011). Here, we will focus on specific vascular features of the V-SVZ.

Dividing progenitor cells (type B cells) and their transit-amplifying type C cells lie adjacent to the extensive planar vascular plexus in the V-SVZ. Approximately 47% of dividing type B cells and 46% of type C cells are found within five microns of the vasculature. During homeostasis and regeneration, type B cells and type C cells directly contact SVZ BV sites devoid of astrocyte end-feet and pericyte coverage (Shen et al., 2008; Tavazoie et al., 2008). Most dividing type B and type C cells are close to these sites, highlighting the importance of vasculature in supporting progenitor cell function. By contrast, most migrating neuroblasts are more distal to the vasculature (only 14% are within 5 µm) compared to type B and type C cells, even though BV run parallel to the aggregates of migrating neuroblasts chains in the dorsal aspect of the SVZ and in the RMS. However, it still remains to be understood whether neuronal differentiation occurs in response to leaving the vascular bed or whether cells leave the vasculature after they are differentiated (Tavazoie et al., 2008).

BV in the V-SVZ region also serve as a scaffold for long-distance migration of neuroblasts from V-SVZ to the olfactory bulb, potentially through the release of chemoattractant (e.g., BDNF, vascular endothelial growth factor (VEGF)) and chemorepulsive factors (e.g., semaphorins, ephrins) (Bovetti et al., 2007; Snapyan et al., 2009; Whitman et al., 2009; Kojima et al., 2010). Migrating neuroblasts are ensheathed by a layer of astrocyte processes and use each other as guides in the migration process toward the olfactory bulb. Similarly, in animal stroke models (Ohab et al., 2006) and human stroke patients (Jin et al., 2006), a long-distance migration of newly born immature neurons from SVZ to peri-infarct cortex is observed. Stromal derived factor-1/C-X-C chemokine receptor 4 (SDF-1/CXCR4) signaling assists BV- and astrocyte-associated migration of adult SVZ progenitors after cortical injury (Saha et al., 2013). Recent studies have identified that SDF-1/CXCR4-mediated signaling is a critical homing factor in the V-SVZ niche. CXCR4 is expressed by all progenitor cells in the SVZ. SDF1 is expressed in the SVZ BV, but the ependymal cells that line the lateral ventricles express higher level of SDF-1 to create a concentration gradient. SDF1 increases integrin *α*6 and epidermal growth factor receptor (EGFR) expression in activated type B and type C cells, enhancing their activated state and ability to bind laminin in the vascular niche (Kokovay et al., 2010). SDF1 also increases the motility of type A neuroblasts. These type A cells express lower levels of integrin *α*6, which might promote evacuating from the vascular niche.

As noted, beside the role as a conduit for blood delivery for brain, cerebral BV (cerebral endothelial cells) support neighboring cells by secreting trophic factors. Recent studies confirmed that cerebral endothelial cells regulate the function of oligodendrocyte lineage cells. In co-culture system of endothelium with NSPCs, the chemokine C-C motif chemokine 2/monocyte chemotactic protein-1 (CCL2/MCP-1) mediates the interaction between endothelium and neural precursor cells to promote the differentiation of NSPCs into oligodendrocytes (Chintawar et al., 2009). Another study used the *in vitro* media-transfer system to show that conditioned medium from endothelial cells promotes the differentiation of NSPCs into oligodendrocyte lineage cells (Plane et al., 2010). In addition, cerebral endothelial cells and OPCs may provide an oligovascular niche to promote the proliferation and migration of OPCs (Arai and Lo, 2009a; Hayakawa et al., 2011, 2012). This endothelium-to-OPC supportive signaling would be attenuated by excessive oxidative stress (Arai and Lo, 2009b), supporting the idea that oligodendrocyte/myelin maintenance and renewal is disturbed under pathological conditions.

### Extracellular matrix (ECM)

The vascular and extravascular basal lamina (BL) are composed of the ECM proteins such as laminin, heparan sulfate proteoglycans (HSPG), and collagen-IV. The BL determine inductive microenvironments for adjacent stem cells by providing, storing, and compartmentalizing growth factors and cytokines. These factors can be concentrated in the extravascular BL and bind to cellular receptors present on the cells in direct contact with the BL (Roberts et al., 1988; Yayon et al., 1991). The extravascular BL are continuous with the surrounding local BV (i.e., vascular BL). However, the BL project into the V-SVZ independently from BV and terminate underneath the ependyma. Notably, compared with the cellular volume in the V-SVZ, the V-SVZ extravascular BL occupy a smaller volume, but are folded, branched, and fractionated to increase contact surface with the cellular environment. As these features are characteristics of a fractal structure, the V-SVZ extravascular BL was termed “fractone” (Mercier et al., 2002). Anatomically, the fractone is very efficient in contacting an enormous number of cells in the wall of the ventricle. Therefore, this structure may help V-SVZ progenitors receive blood/CSF-borne information from virtually all brain sites, circumventricular organs, and peripheral organs.

ECM proteins themselves are also active molecules for V-SVZ progenitor cells. Mice lacking laminin *α*2 subunit (LAMA2-/-) have fewer OPCs both in the dorsal SVZ and an adjacent developing white matter tract, coupled with high levels of OPC death (Relucio et al., 2012). Furthermore, defects in the spatial organization of IPCs in the perinatal V-SVZ niche lead to defective oligodendrocyte maturation and myelination (Relucio et al., 2012). These findings indicate that laminin promotes the survival of OPCs in the gliogenic niche, allowing the appropriate numbers of OPCs to populate their target white matter tracts. This survival-promoting effect is partly due to localizing or enhancing trophic factor signals.

Taken together, fractones, perivascular, and subpial BL constitute an ideal anatomic mechanism for exchanging growth factors and cytokines between extraparenchymal and NSPCs in the V-SVZ. This environment may also prevent extensive diffusion of these signaling molecules in the extracellular environment.

### Cerebrospinal fluid (CSF)

Type B cells in the V-SVZ extend an apical primary cilium toward the brain ventricular space. The space is filled with CSF and the composition of CSF can modulate the self-renewal, proliferation, and differentiation of V-SVZ progenitor cells (Falcao et al., 2012). CSF is secreted mainly from the choroid plexus (CP), located in the caudal regions of the lateral ventricle. The adult CP expresses and secretes numerous trophic factors and cytokines, which could influence the dynamics of V-SVZ progenitor cells (Falcao et al., 2012). For example, CP-secreted IL-1*β* binds to IL-1 receptors on the surface of type B cells to upregulate vascular cell adhesion molecule 1 (VCAM1) expression. This change promotes the adhesion of type B cells to the neural stem cell niche and the pinwheel architecture of ependymal cell rosettes via maintenance of redox homeostasis by NADPH oxidase 2 (NOX2) activation. In turn, inhibition of VCAM1 stimulates quiescent Type B cells to proliferate and advance through the cell lineage to type A neuroblasts that migrate to the olfactory bulb (Kokovay et al., 2012).

Factors in CSF or CP may also affect V-SVZ progenitor cells even under pathological conditions. In a Lysophosphatidylcholine (LPC)-induced demyelination model, intraventricular infusion of epidermal growth factor (EGF) dramatically promoted the proliferation and migration of SVZ NSPCs as well as their differentiation into oligodendrocytes (Gonzalez-Perez et al., 2009). Intraventricular infusion of the bone morphogenetic protein (BMP) inhibitor Noggin also increased the number of Olig2-positive oligodendrocytes after cuprizone-induced demyelination in mice (Cate et al., 2010). Additionally, CP is also a source of chemorepulsive factors, including Slits, Semaphorins, and ephrins, which can influence V-SVZ NSPCs migration. For instance, the infusion of the Ephrin-B2 ligand in the lateral ventricles disrupts the migratory chain of neuroblasts and increases the proliferation of type B cells. Another report has shown that the ciliary beating of ependymal cells in the wall of lateral ventricle generates CSF flow, which forms a concentration gradient of chemoattractants secreted by the CP. Such guidance molecules gradients may contribute to the directional migration of neuroblasts to the olfactory bulbs (Sawamoto et al., 2006).

## Intrinsic factors that promote oligodendrocyte generation

### Transcriptional factors

A dynamic combination of transcription factors may modulate oligodendrocyte maturation (Nicolay et al., 2007). The different stages of oligodendrocyte development (specification, proliferation, differentiation, and myelination) are spatially and temporally regulated by various transcription factors under the control of multiple signaling pathways, such as Wnt (Fancy et al., 2009), sonic hedgehog (Shh; Lu et al., 2000), BMP (Samanta and Kessler, 2004; Jablonska et al., 2010), and Notch (Wang et al., 1998; Nicolay et al., 2007). In this section, we will overview key transcription factors that regulate the function of oligodendrocyte lineage cells.

The basic helix-loop-helix (bHLH) transcription factors Olig1 and Olig2 have been extensively studied in oligodendrocyte development (Lu et al., 2002; Zhou and Anderson, 2002). An *in vivo* gain-of-function study has shown that the inducible overexpression of Olig2, but not Olig1, in SVZ progenitor cells increases the generation of OPCs. These newly generated OPCs migrate and differentiate into mature oligodendrocytes in the corpus callosum, cortex, and olfactory bulb. Subsequently, these cells lead to precocious myelination with an increase in the number of astrocytes in the corpus callosum at postnatal CNS myelination stage (Maire et al., 2010). Olig2-expressing cells may represent a transition state between type B and C cells. Olig2 over-expression directs SVZ progenitors towards oligodendrocyte and astrocyte fates, while it opposes the neurogenic role of Pax6 and represses neuronal lineages (Hack et al., 2005; Marshall et al., 2005). In addition, the interactions between Olig and Id proteins have been reported to mediate the inhibitory and promoting effects of BMP4 on oligodendrogenesis and astrogliogenesis, respectively (Samanta and Kessler, 2004).

Many other factors have also been reported to regulate oligodendrocyte specification and development (Nicolay et al., 2007). Firstly, an oligodendrocyte-specific zinc finger transcription repressor Zfp488, a downstream effector of Olig1, favors oligodendrocyte maturation in concert with Olig2 (Wang et al., 2006). Retrovirus-mediated Zfp488 overexpression in SVZ NSPCs can increase the number of oligodendrocytes in the corpus callosum and leads to functional recovery after cuprizone-induced demyelination in mice (Soundarapandian et al., 2011). Secondly, a proneural transcription factor Ascl1/Mash1 operates in genetic interaction with Olig2 during OPC specification in the embryonic telencehpalon and the loss of Ascl1 reduces embryonic oligodendogenesis (Parras et al., 2007; Sugimori et al., 2007). Recent conditional deletion and lineage tracing study has demonstrated that Ascl1 positively regulates OPC specification from SVZ progenitors. The study also shows that Ascl1 controls the proper differentiation into oligodendrocytes during postnatal myelination stage and remyelination after LPC-induced demyelination (Nakatani et al., 2013). In parallel with the above findings, postmortem examination of human periventricular MS lesions confirmed that Ascl1 expression is a hallmark of OPCs involved in myelin repair (Nakatani et al., 2013). In addition, Ascl1 induces Notch-mediated repression of the neurogenenic determinants Dlx1/2, which may promote oligodendrogenesis at the expense of an astrocytic fate (Nakatani et al., 2013). Thirdly, members of the SRY-box (Sox) transcription factors have also emerged as crucial regulators of oligodendrocyte behavior. Sox8, 9, and 10 induce early postnatal SVZ NSPCs toward the oligodendrocyte lineage fate (Pozniak et al., 2010), while Sox4, 5, and 6 have inhibitory roles in timing oligodendrocyte specification and terminal differentiation (Potzner et al., 2007). The gain-of-function approach has shown that Sox17 overexpression in oligodendrocyte lineage cells promotes postnatal oligodendogenesis and prevents cell loss after LPC-induced demyelination by increasing oligodendrocyte lineage cells (Ming et al., 2013). Another study has also demonstrated that the suppression of Wnt/*β*-catenin signaling by Sox17 enhances progenitor cell maturation (Chew et al., 2011). Additionally, nuclear factor 1A (NF1A) NF1A is expressed in OPCs, but not in mature oligodendrocytes during mouse embryonic development. Similarly, NF1A is observed in only OPCs in demyelinated white matter lesions of human neonatal hypoxic-ischemic encephalopathy (HIE) or adult MS. During development or neonatal/adult remyelination after injury, NF1A suppresses OPC differentiation via direct repression of myelin gene expression (Fancy et al., 2012). The role of NF1A on OPCs during remyelination is a recapitulation of development (Fancy et al., 2012). These findings may indicate that downregulation of NF1A stimulates OPC differentiation while deregulation of NF1A contributes to the suppression of remyelination in white matter disorders.

Taken together, various transcription factors combined with multiple other cofactors and signaling pathways lead to the determination of cell fate under developmental stage and during post-injury remyelination. In turn, factors that regulate oligodendrocyte lineage cells can exert the opposing effects for the fate of neuronal and astroglial lineage cells. It still remains largely unknown how niche-provided signals modulate transcription factor expression. However, the mechanisms and expression patterns of transcription factors during developmental myelination may have some similarities with those during remyelination after myelin loss or oligodendrocyte death.

### Epigenetic modulators

During development, the crosstalk between transcription factors and epigenetic modulators of gene expression is essential for the acquisition of specific cell fates (Hemberger et al., 2009). The epigenetic regulation also influences the multiple steps of oligodendrocyte generation (Liu and Casaccia, 2010); i.e., from embryonic stem cells to OPCs via multipotential neural precursors or even from OPCs to myelinating oligodendrocytes. The epigenetic modulators represent post-translational modifications of nucleosomal histones, changes in histone variants, chromatin remodeling enzymes, DNA methylation, and microRNAs (miRNAs). Among them, we will mainly focus on histone deacetylases (HDACs) and miRNAs in this section.

Persistent histone acetylation in OPCs alters their lineage choice decision by suppressing the acquisition of the oligodendroglial identity. The histone acetylation also favors a conformation of chromatin that is consistent with the establishment of a neuronal or astroglial phenotype (Liu et al., 2007). The oligodendrocyte identity of OPCs is dependent on HDAC enzymatic activity. When HDAC is high, the epigenetic memory of specified progenitors in oligodendrocyte is established by repressing neuronal and astroglial genes. By contrast, when HDAC activity is inhibited, the progenitors are unable to establish an oligodendrocyte-specific program of gene expression and as a response to neurogenic or astrogliogenic signals they are reprogrammed into a multipotential state (Liu et al., 2007). Hence, HDAC inhibition may erase the epigenetic memory of oligodendrocyte in the progenitor cells. In turn, the HDAC inhibition allows the cells to acquire a pattern of gene expression consistent with neuronal and astroglial lineage. In accordance with this phenomenon, global histone acetylation is detected in precursor cells during the early stages of brain development, which are associated with neurogenesis and astrogliogenesis. In contrast, histone deacetylation prevails in OPCs during the later stages of postnatal development and coincides with the onset of myelination (Shen et al., 2005). Furthermore, a genetic ablation loss-of-function study has shown that HDAC1 and HDAC2 are required for oligodendrocyte differentiation (Ye et al., 2009). HDAC1/2 also controls the Wnt signaling pathway, which is known as an inhibitory signal for oligodendrocyte differentiation (Ye et al., 2009). Notably, some extrinsic factors can regulate oligodendrocyte differentiation, at least in part by modifying histone acetylation. For example, Shh induces histone deacetylation via HDACs to promote oligodendrocyte differentiation, while BMP4 blocks the deacetylation and inhibits oligodendrogenesis (Wu et al., 2012). Therefore, one of the major roles of HDACs may repress certain gene expressions that normally blocks OPC differentiation thus allowing NSPCs to mature into myelinating oligodendrocytes (Zuchero and Barres, 2013).

miRNAs are also important epigenetic regulators of various aspects of CNS development and homeostasis by responding to environmental cues and cellular states. miRNAs have an advantage over mRNAs as they are more stable (Jung et al., 2010). A number of miRNAs have been recently shown to play a critical role in oligodendrogenesis, i.e., cell proliferation, differentiation, and myelin formation (Barca-Mayo and Lu, 2012). For example, miR-219 and miR-338 are increased at the onset of oligodendrocyte myelination and play a positive role in the OPC differentiation to mature oligodendrocytes (Dugas et al., 2010; Zhao et al., 2010). miR-219 and miR-338 suppress the expressions of platelet-derived growth factor receptor *α* (PDGFR*α*), hairy and enhancer of split 5 (Hes5), and Sox6 that are known to inhibit OPC differentiation and to maintain OPC in the proliferative state (Dugas et al., 2010; Zhao et al., 2010). The two miRNAs also inhibit Zfp238, FoxJ3 and NeuroD1, which shift the fate of NSPCs from OPCs to neuron lineage (Dugas et al., 2010; Zhao et al., 2010). Thus, the interplay of transcription factors and epigenetic modifiers should be required for the precise regulation of the NSPC-to-oligodendrocyte transition.

## Oligodendrogenesis after white matter damage

**Figure 3:**
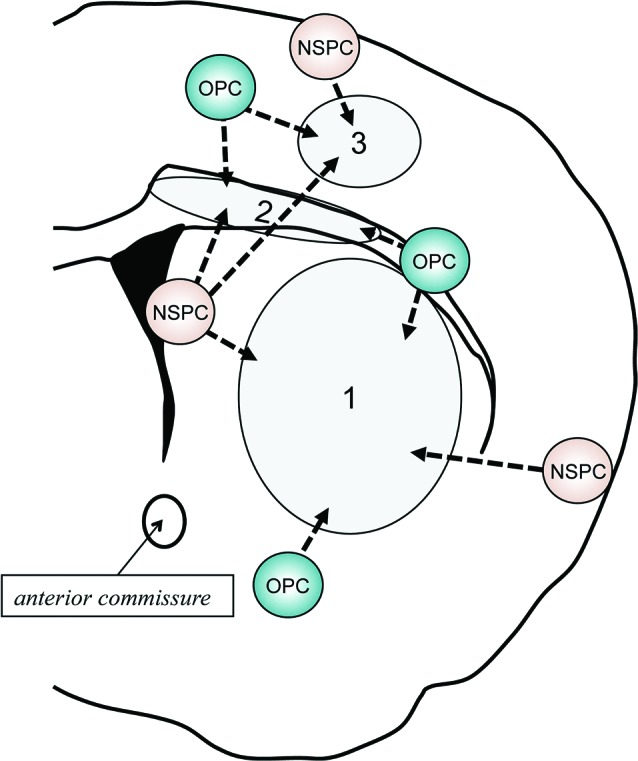
**Schematic of behavior of NSPCs and OPCs after demyelination**. In response to myelin loss or oligodendrocyte death, both NSPCs and OPCs would attempt to repair the white matter damage by proliferating, migrating to the injured areas, and restoring myelinating oligodendrocytes. If the damaged area is restricted in the corpus callosum (2), V-SVZ-derived NSPCs would shift from neuronal lineage cells to oligodendroglial lineage cells. In addition, residing OPCs adjacent to the damaged area may also contribute to the repairing. Although the V-SVZ-derived NSPCs could travel to the cortex (1) or striatum (3), the recruitment of local OPCs/NSPCs outside of the V-SVZ (e.g., pia matter and/or cortical layer 1) to the lesion area would be more important when the demyelination occur in these areas.

Both NSPCs in V-SVZ and OPCs outside of V-SVZ exhibit endogenous repair attempts in response to demyelination (Menn et al., 2006; Figure 3). In this section, we will overview oligodendrocyte regeneration attempts in human and small animals, focusing on the pathophysiological conditions in MS and vascular dementia.

### Multiple sclerosis (MS)

MS is characterized by inflammation, demyelination, and axonal damage in the CNS with different degrees of autoimmune involvement (Sospedra and Martin, 2005; Fugger et al., 2009). The typical disease course after the first attack consists of remissions and relapses with slow onset of disability (Fugger et al., 2009). In general, the extent of remyelination varies from patient to patient and from lesion to lesion. The remyelination attempt is mostly restricted to a thin rim around the lesion edge and decreases as the disease progresses. However, non-negligible remyelination may occur after white matter damage in MS patients. Post-mortem studies have revealed that the density of glial fibrillary acidic protein (GFAP)-positive astrocytes and early progenitors in the SVZ is increased in MS patients. In the subependymal region, these progenitors express early glial markers such as Sox9, Sox10, and Olig2. Similar progenitors prevail in periventricular lesions. The polysialylated neuronal cell adhesion molecule (PSA-NCAM) is a marker for developing and migrating neuronal progenitors in the immature vertebrate nervous system. The presence of PSA-NCAM-positive progenitors with a bipolar morphology in the lesion area may suggest their potential migration within or away from the SVZ to oligodendrocyte renewal/repairing (Nait-Oumesmar et al., 2007). As mentioned, remyelination attempts often fail in chronic MS patients. While the underlying mechanisms are still mostly unknown, the failure might correspond to reduced recruitment and/or disturbed maturation of OPCs (Nait-Oumesmar et al., 2008; Kotter et al., 2011). Recently, TAT-interacting protein 30 kDa (TIP30), a proapoptotic factor, was proposed as a new pathogenic factor in MS. In human chronic MS lesions, Notch1 is activated in OPCs and Contactin is abundantly expressed on demyelinated axons. This noncanonical pathyway (Notch1-F3/Contactin) is important for OPC differentiation and axon myelination. The pathologic upregulation of TIP-30 blocks the nuclear transport of Notch1 intracellular domain, thus leading to disruption of the noncanonical pathyway (Nakahara et al., 2009).

Analysis of lesion-induced oligodendrogenesis in experimental rodent models would be needed to design repair strategies in white matte-related diseases (Keough and Yong, 2013). There are several animal models of MS, including EAE model, targeted EAE (tEAE) model, and cuprizone model. In most animal models of MS, activation of the V-SVZ (i.e., increase in the number of SVZ-NSPCs) is confirmed, and NSPCs in the V-SVZ have been shown to migrate and undergo oligodendrogenesis in demyelinated lesions (Picard-Riera et al., 2002). In addition, remyelination failure in the MS models is not attributed to an absence or a reduction of OPCs in the lesion area. Rather, the failure is a result of the lack of positive signals for oligodendrocyte maturation/myelination or the overactivation of inhibitory signals from immune cells for the myelination program (Back et al., 2005; Sloane et al., 2010; Kotter et al., 2011). However, under some conditions, endogenous microglia and infiltrating macrophages would work for promoting the oligodendrocyte remodeling/repairing (Napoli and Neumann, 2010). The myelin debris is generated during demyelination and the containing proteins inhibit OPC differentiation, but microglia and macrophages try to remove the myelin debris. These immune cells also secrete soluble mediators, which attract the phagocytic and repair-promoting effectors. In addition, TNF-*α* dearth may lead to a significant delay in remyelination with a reduction of proliferation and maturation of OPCs in mouse MS models. Analysis with mice lacking TNF-R1 or TNF-R2 has demonstrated that TNF-*α* signaling through TNF-R2 promotes the accumulation of proliferating OPCs (Arnett et al., 2001). Furthermore, a transcriptomic analysis in a mouse cuprizone model of MS reveals that microglia can exhibit the phenotype of supporting remyelination. These microglia produce a rich repertoire of cytokines and chemokines to recruit endogenous OPCs to the lesion site for repairing the damaged myelin sheathes (Olah et al., 2012).

Small animal models are useful to examine the precise mechanisms of oligodendrogenesis after white matter injury. However, it should be noted that behavior of NSPCs (lineage commitment, migration, maturation/myelination) and glial activation are different among animal models. For example, compared to myelin oligodendrocyte glycoprotein (MOG)-induced EAE model in C57BL/6, a model using SJL mice shows persistent activation of microglia in the forebrain, which is similar to current observations in the cortex of MS patients (Kutzelnigg et al., 2005; Rasmussen et al., 2007). In this model, NSPCs proliferate and engage in repair during the acute phase of EAE, but this capacity is lost during the chronic phase of the disease. As the number of microglia is in an inverse relationship with the proliferative activity of SVZ cells in the SJL model, chronic microglial activation in the SVZ may have a tonic inhibitory role on NSPC proliferation (Rasmussen et al., 2011). Ultimately, preclinical studies are required to be conducted in multiple animal models of MS.

### Vascular dementia

Although not typically thought of as a demyelinating disease, white matter injury comprise a critical part of vascular dementia pathophysiology. Vascular dementia accounts for 20% of the 25 million people with dementia worldwide. It is also increasingly recognized that Alzheimer’s disease and vascular dementia may belong to a continuous spectrum of diseases based on vascular pathologies (Viswanathan et al., 2009). In addition, 25% of older stroke patients develop dementia within 3 months of a stroke (Censori et al., 1996), and there is a 10-fold increased risk of delayed dementia over the subsequent 5 years in stroke survivors compared to people of the same age (Kokmen et al., 1996). Postmortem human brain analyzes have demonstrated a significant increase of progenitor cells with nestin, PSA-NCAM, and Dcx expression in the SVZ and peri-infarct regions in vascular dementia patients (Ekonomou et al., 2011). Another postmortem human brain study has shown that in ischemic white matter lesions of vascular dementia patients, OPCs were increased, but oligodendocytes were decreased (Miyamoto et al., 2010). These studies may suggest that to some extent the endogenous regenerative attempts in oligodendrogenesis occur in the brain of vascular dementia patients. However in most cases, complete recovery of function cannot be achieved, probably due to various inhibitory factors related to chronic ischemic lesions. The detailed mechanisms that suppress oligodendrocyte regeneration in patients with vascular dementia still remain unclear. However, recent efforts using rat or mouse models of vascular dementia have proposed some promising cues in understanding the pathophysiology of vascular dementia.

Vascular dementia is characterized by cognitive impairment, cerebrovascular white matter changes, and cerebral hypoperfusion. In this regard, rat and mouse models of prolonged cerebral hypoperfusion have been developed and widely used (Farkas et al., 2007; Ihara and Tomimoto, 2011). The rat model is accompanied with cognitive impairment and cholinergic deficits (Wakita et al., 1994; Ni et al., 1995). These animals develop demyelination with axonal damage (Wakita et al., 2002), which appears similar to that found in human cerebrovascular white matter lesions. This model also shows an increase of OPCs in the demyelinating lesions (Miyamoto et al., 2010; Chida et al., 2011). The extent of demyelination is in inverse correlation with cognitive function (Chida et al., 2011), and therefore treatments that can enhance remyelination may ameliorate the cognitive dysfunction under prolonged cerebral hypoperfusion. A mouse model of prolonged cerebral hypoperfusion is achieved by narrowing the bilateral common carotid arteries (CCAs) with newly designed micro-coils (Shibata et al., 2004, 2007). This model demonstrates good reproducibility of the white matter changes seen in clinic, including blood-brain barrier disruption, glial activation, oxidative stress, and oligodendrocyte loss. In this mouse model, the cerebral white matter is selectively damaged, and the integrity of the gray matter (including hippocampal) remains intact at a month after the surgery if the bilateral CCAs are appropriately placed by 0.18 mm internal diameter micro-coils (Shibata et al., 2004, 2007). Recent study using this model has demonstrated that after induction of prolonged cerebral hypoperfusion, the numbers of newborn oligodendrocytes and their precursors are transiently increased in 2-month old mice (Miyamoto et al., 2013a). On the contrary, these endogenous repairing attempts are significantly dampened in older mice (8-month old) partly due to defects in cyclic AMP response element-binding protein (CREB) signaling (Miyamoto et al., 2013a). In fact, activating CREB signaling by the treatment of phosphodiesterase (PDE)-III inhibitor cilostazol increased the oligodendrogenesis in the older mice (Miyamoto et al., 2013a). More recently, another study using the mouse model has shown that excessive oxidative stress under prolonged cerebral hypoperfusion may disrupt the differentiation from OPCs to oligodendrocytes (Miyamoto et al., 2013b). These findings may suggest that drugs that promote oligodendrocyte regeneration can be useful for vascular dementia patients. To note, effects of those drugs on white matter remodeling after injury should be carefully examined in preclinical studies before testing them in clinical trials. In addition, further studies are also warranted to elucidate the regulatory mechanisms in NSPCs-to-OPC transition under cerebral hypoperfusion conditions.

## Conclusion

The V-SVZ region possesses the specialized microenvironments that enable NSPCs to have efficient and dynamic interactions with the V-SVZ components. The fate of NSPCs under physiological conditions is tightly regulated by the combined actions of intrinsic and extrinsic factors. After demyelination, the balance would be changed to promote the endogenous repairing attempts in oligodendrogenesis. On the other hand, as the disease conditions progress, the NSPC-OPC-oligodendrocyte transition becomes disrupted, mainly due to the decrease of pro-oligodendrogenesis signals and the increase of anti-oligodendrogenesis signals. A deeper understanding of the underlying mechanisms in oligodendrocyte generation from NSPCs may lead us to effective therapeutic approaches for white matter related diseases. But it should be noted that factors that regulate the fate of NSPCs sometimes exhibit opposing effects between neurogenesis and gliogenesis/oligodendrogenesis. Ultimately, we may need to consider the balance between neurogenesis and gliogenesis/oligodendrogenesis in pursuing therapeutic strategies for ameliorating white matter damage and dysfunction in CNS disease.

## Conflict of interest statement

The authors declare that the research was conducted in the absence of any commercial or financial relationships that could be construed as a potential conflict of interest.
